# Human metabolism and kinetics of the UV absorber 2-(2H-benzotriazol-2-yl)-4,6-di-tert-pentylphenol (UV 328) after oral administration

**DOI:** 10.1007/s00204-021-03093-1

**Published:** 2021-06-27

**Authors:** Heike Denghel, Julia Hiller, Edgar Leibold, Thomas Göen

**Affiliations:** 1 Institute and Outpatient Clinic of Occupational, Social and Environmental Medicine, Friedrich-Alexander-Universität Erlangen-Nürnberg, Henkestraße 9-11, 91054 Erlangen, Germany; 2 BASF SE, Product Safety, Carl-Bosch-Straße 38, 67056 Ludwigshafen am Rhein, Germany

**Keywords:** UV 328, UV absorber, Oral application, Kinetics study, Human biomonitoring

## Abstract

2-(2*H*-Benzotriazol-2-yl)-4,6-di-*tert*-pentylphenol (UV 328; CAS: 25973-55-1) is an ultraviolet light (UV) absorber which belongs to the class of hydroxy phenol benzotriazoles. Therefore, UV 328 is added to plastics and other polymers due to its photostability to prevent discoloration and prolong product stability which may result in an exposure of consumers. However, information about the toxic effects on humans and the human metabolism are still lacking. In the present study, human metabolism pathways of UV 328 and its elimination kinetics were explored. For that purpose, three healthy volunteers were orally exposed to a single dose of 0.3 mg UV 328/kg bodyweight. UV 328 and its metabolites were investigated in blood and urine samples collected until 48 and 72 h after exposure, respectively. Thereby, previously published analytical procedures were applied for the sample analysis using dispersive liquid–liquid microextraction and subsequent measurement via gas chromatography coupled to tandem mass spectrometry with advanced electron ionization. UV 328 was found to be oxidized at its alkyl side chains leading to the formation of hydroxy and/or oxo function with maximum blood concentrations at 8–10 h after exposure for UV 328-6/3-OH, UV 328-4/3-OH and UV 328-4/3-CO. In contrast, a plateau for UV 328-4/3-CO-6/3-OH levels was reached around 10 h post-dosage. The highest blood levels were found for native UV 328 at 8 h after ingestion. Furthermore, biphasic elimination kinetics in blood were revealed for almost all detected metabolites. UV 328 and its metabolites did not occur in blood as conjugates. The renal elimination kinetics were very similar with the kinetics in blood. However, the prominence of the metabolites in urine was somewhat different compared to blood. In contrast, mostly conjugated metabolites occurred for renal elimination. In urine, UV 328-4/3-CO-6/3-OH was found to be the most dominant urinary biomarker followed by UV 328-6/3-OH and UV 328-4/3-OH. In total, approximately 0.1% of the orally administered dose was recovered in urine within 72 h. Although high levels of UV 328 in blood proved good resorption and high systemic availability of the substance in the human body, the urine results revealed a rather low quantitative metabolism and urinary excretion rate. Consequently, biliary excretion as part of the enterohepatic cycle and elimination via feces are assumed to be the preferred pathways instead of renal elimination.

**Supplementary Information:**

The online version contains supplementary material available at 10.1007/s00204-021-03093-1.

## Introduction

**Fig. 1:**
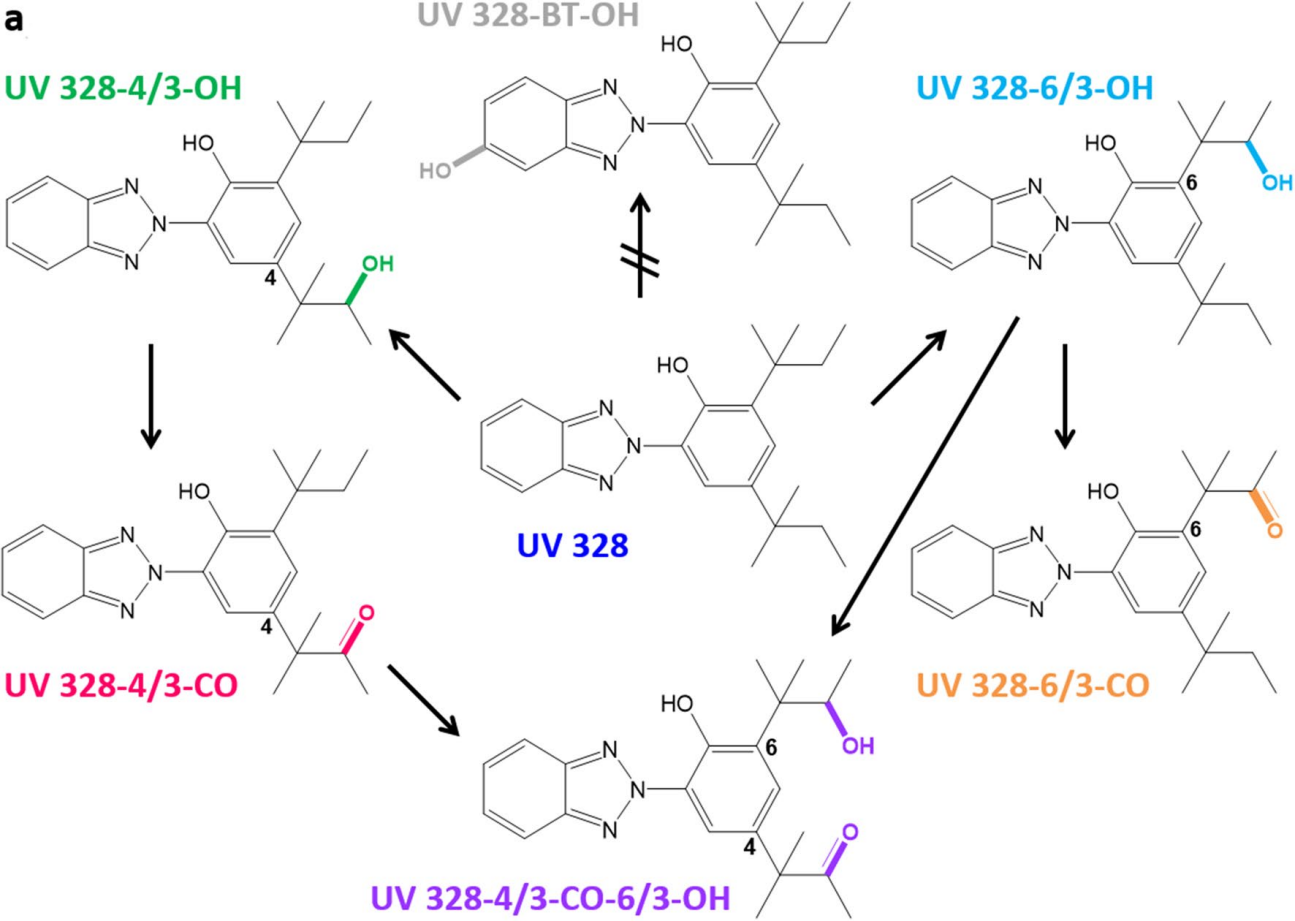
Predicted metabolism of UV 328. Confirmed metabolites: 2-(2*H*-benzotriazol-2-yl)-4-(2-methylbutan-3-on-2-yl)-6-(3-hydroxy-2-methylbutan-2-yl)phenol (UV 328-4/3-CO-6/3-OH); 2-(2*H*-benzotriazol-2-yl)-6-(3-hydroxy-2-methylbutan-2-yl)-4-(*tert*-pentyl)phenol (UV 328-6/3-OH); 2-(2*H*-benzotriazol-2-yl)-4-(3-hydroxy-2-methylbutan-2-yl)-6-(*tert*-pentyl)phenol (UV 328-4/3-OH); 2-(2*H*-benzotriazol-2-yl)-4-(2-methylbutan-3-on-2-yl)-6-(*tert*-pentyl)phenol (UV 328-4/3-CO); 2-(2*H*-benzotriazol-2-yl)-6-(2-methylbutan-3-on-2-yl)-4-(*tert*-pentyl)phenol (UV 328-6/3-CO). Unconfirmed metabolite: 2-(2*H*-5-hydroxybenzotriazol-2-yl)-4,6-di-(*tert*-pentyl)phenol (UV 328-BT-OH)

2-(2*H*-Benzotriazol-2-yl)-4,6-di-*tert*-pentylphenol (UV 328; CAS: 25973-55-1) (see Fig. 1) is an ultraviolet light (UV) absorber which belongs to the class of hydroxy phenol benzotriazoles. Therefore, UV 328 is added to plastics and other polymers due to its photostability to prevent discoloration and prolong product stability (Wypych and Wypych 2015). As a result, UV 328 is used as a UV protection agent in plastics, rubber, resins and cosmetics as well as in coatings for e.g. cars, wood and textiles (ECHA 2018, 2021b). Furthermore, UV 328 is added to adhesives, sealants, coatings, paints, thinners, paint removers and polymer preparations and compounds. UV 328 is recommended for plastics such as polyethylene, polyvinyl chloride, polyurethane, polymethyl methacrylate and polypropylene (Jia et al. 2007; Wypych and Wypych 2015). According to its industrial uses, UV 328 is expected to be released to surface waters via industrial effluent as well as from the disposal of products degrading and releasing the substance (Government of Canada 2016). As this chemical is often not fully removed during waste water treatment (Lu et al. 2017; Montesdeoca-Esponda et al. 2015; Song et al. 2014), UV 328 can enter the environment via drain water and sewage sludge (Carpinteiro et al. 2012b; Casado et al. 2013; Montesdeoca-Esponda et al. 2012, 2013b; Nakata and Shinohara 2010; Ruan et al. 2012; Zhang et al. 2011). Thus, this substance was also detected in environmental water samples (Carpinteiro et al. 2010a; García-Guerra et al. 2016; Kameda et al. 2011; Liu et al. 2014; Montesdeoca‐Esponda et al. 2013a; Vimalkumar et al. 2018), biosolid-amended soils (Lai et al. 2014) and sediments (Apel et al. 2018a, b; Cantwell et al. 2015; Carpinteiro et al. 2012a; Chiaia-Hernandez et al. 2013; Kameda et al. 2011; Mizukawa et al. 2017; Montesdeoca‐Esponda et al. 2013b; Nakata et al. 2009; Peng et al. 2017b; Reddy et al. 2000; Wick et al. 2016). Due to its resistance to environmental degradation through natural chemical, biological and photolytic processes, UV 328 was identified as a persistent, bioaccumulative and toxic substance (PBT) under the criteria of REACH and is therefore listed as a “substance of very high concern” (SVHC) (Brandt et al. 2016; ECHA 2021b). Additionally, plastic fragments and marine debris from beaches may cause an exposure of seabirds and other living beings (Rani et al. 2017; Tanaka et al. 2020a). Consequently, the presence of UV 328 was proven in birds, fish, mammals, mussels and other aquatic organisms (Gimeno-Monforte et al. 2020; Kim et al. 2011b; Langford et al. 2015; Lu et al. 2016, 2019; Montesdeoca-Esponda et al. 2020; Nakata et al. 2010, 2012; Peng et al. 2017a, 2015, 2020; Pruell et al. 1984; Tanaka et al. 2019, 2020b). It was further found in indoor dust (Carpinteiro et al. 2010b; Kim et al. 2012), textiles (Avagyan et al. 2015) and human breast milk (Kim et al. 2019).

No acute toxicity was observed for oral exposure in animal studies (CIBA-GEIGY 1978; Kim et al. 2011a). In contrast, specific target organ toxicity to liver and kidneys was stated for repeated oral administration to rats and beagle dogs (ECHA 2013; Geigy 1970; Til et al. 1968). Chronic exposure of fish induced oxidative stress in the liver so that significant effects to antioxidant enzymes as well as tissue damage were reported (Hemalatha et al. 2020). Furthermore, accumulation of UV 328 in fish liver was found after chronic food-borne exposure together with transcriptional changes in gene expression (Giraudo et al. 2020). Thereby, induced transcription of ribosomal protein was observed together with the downregulation of genes involved in immune responses. However, little is known about the human toxicology and metabolism of this substance. In view of its persistence on the one hand and the lack of human data on the other hand, the substance was selected in the joint human biomonitoring program of the Federal Ministry for Environment, Nature Conservation and Nuclear Safety (BMU) and the Association of the German Chemical Industry (VCI) (Kolossa-Gehring et al. 2017).

First insight in the human metabolism of UV 328 was gained by in vitro studies using human liver microsomes (HLM) (Denghel et al. 2019; Zhuang et al. 2017). Zhuang et al. (2017) postulated three mono- and three dihydroxylated metabolites of UV 328 applying a non-target approach with Cytochrome P450 3A4 and HLM. Denghel et al. (2019) identified further oxidation products using suspect screening analysis and verified five of the six investigated compounds in a target approach after the custom synthesis of the postulated metabolites. Thereby, oxidation of UV 328 at one or both alkyl side chains resulting in hydroxy and/or oxo-metabolites was proven. However, metabolization at the benzotriazole moiety was not confirmed (Denghel et al. 2019).

For the further elucidation of the human metabolism of UV 328, analytical procedures for the determination of UV 328 and its metabolites in human blood and urine were developed, optimized and validated in a first step (Denghel and Göen 2020, 2021). The aim of the presented in vivo study was investigating the metabolism and kinetics of UV 328 in humans and exploring options of human biomonitoring for the compound.

## Materials and methods

### Study design

Three volunteers (two men and one woman, mean age 37.3 ± 15.5, mean body weight 81.3 ± 6.1 kg) were selected for the in vivo study. Prior to oral exposure, every volunteer collected one urine sample. Additionally, one blood sample was taken using EDTA-Monovettes^®^ (Sarstedt, Nümbrecht, Germany) before exposure. Subsequently, the volunteers were orally exposed to a dose of 0.3 mg UV 328/kg bodyweight (bw). Therefore, 22.8–26.7 mg (mean 24.5 ± 2.0 mg) UV 328 of highest purity (99.9%) was prepared with butter on a slice of bread and ingested by each volunteer. The doses of exposure were derived from the No Observed Adverse Effect Level (NOAEL) of 30 mg UV 328/kg bw/day for beagle dogs (US EPA 2009). Thereby, a safety factor of 100 was considered for single application and interspecies differences, resulting in a dose of 0.3 mg/kg bw. The volunteers were orally exposed to UV 328 in the early morning and instructed to collect urine samples every hour for the first 10 h at least. Participants collected the total urine volume of each void separately throughout 72 h and recorded the respective sampling times. Altogether, between 34 and 43 urine samples were collected. The total 72 h urine volume ranged from 3.3 to 8.1 L (mean 5.5 ± 2.4 L). The urine samples were volumetrically measured for each sampling point, aliquoted in urine Monovettes^®^ (Sarstedt, Nümbrecht, Germany) and stored frozen at − 50 °C until analysis.

Furthermore, blood samples were drawn from each volunteer 2, 4, 6, 8, 10, 24, 34 and 48 h after exposure. In summary, eight blood samples were collected from every volunteer. One volunteer donated two EDTA—Monovettes^®^ (Sarstedt, Nümbrecht, Germany) for every time point. So, for every sampling time one sample was kept as whole blood while the plasma fraction was extracted for the other. In this case, the respective blood vessels were centrifuged at 800 × *g* for 5 min. The supernatants were transferred to 15 mL conical plastic tubes (Sarstedt, Nümbrecht, Germany). Both blood and plasma samples were stored frozen at − 50 °C until analysis.

The study design was approved by the local ethics committee of the University of Erlangen-Nürnberg (No. 15_16 B). All test persons were informed about the aims and risks of the study and gave their written informed consent to their participation. Furthermore, all volunteers were between 18 and 60 years old, healthy and not occupationally exposed to UV 328. Pregnant and breastfeeding women were excluded from the study. Female participants were obliged to use contraceptives to avoid pregnancy during their participation.

Due to the reported specific target organ toxicity to liver and kidney in animal studies (ECHA 2013; Geigy 1970; Til et al. 1968), standard laboratory values for liver and kidney function [glutamyl oxaloacetic transaminase (GOT), glutamate pyruvate transaminase (GPT), gamma-glutamyltransferase (GGT), alkaline phosphatase (AP), bilirubin, serum creatinine, glomerular filtration rate (GFR), urea] were monitored before, during and one week after oral application to UV 328 for all volunteers. Because of potential confounding effects on those liver and kidney laboratory parameters and their potential to indicate massive cell destruction, lactate dehydrogenase (LDH) and creatinine kinase (CK) were included in the medical blood work as well. All medical laboratory values were carried out by an accredited medical laboratory. Thus, initial pathological laboratory values for liver or kidney function were defined as further exclusion criteria for study participation. In case of occurrence of relevant pathological medical values for liver or kidney function during the ongoing study which could be attributable to the study exposure, no further subjects were to be included until further clarification, potentially leading to a full cessation of the whole study.

The first two volunteers did not show any relevant abnormalities among GOT, GPT, GGT, LDH, bilirubin, urea, albumin, serum creatine and CK at any time. However, after showing no abnormalities of clinical pathological relevance before or 24 h after the exposure, the third subject developed a highly elevated CK value and elevated levels of LDH and GOT seven days after exposure in spite of general well-being of the test person. All other parameters remained within the normal reference. Those abnormalities were further enquired. Subsequent laboratory tests first excluded a myocardial involvement (100% of CK-MM) and showed a steady decline of CK as well as the potentially confounded parameters (LDH, GOT, GPT) over the next days. Causal medication, trauma, known muscular diseases or extreme muscular stress were negated. However, the subject had participated in various moderate sporting activities in the days prior to the day 7 blood drawing. The subject was a generally athletic young male regularly doing sports and indicated no muscle soreness from or extreme exhaustion during the practiced sporting activities. In conjunction of all aspects and consultation with the medical laboratory, the observed abnormalities were interpreted as a sign of isolated damage of skeletal muscles with a corresponding expectable co-reaction of the liver enzymes. This was most likely attributable to the previous sporting activities, although the measured CK value (6784 U/L) was unusually high. From a medical perspective, this effect may also constitute an incidental finding due to an asymptomatic CK hyperemia (Moghadam-Kia et al. 2016). Further follow-up 20 days after the incident abnormalities and without sporting activities during the last week, normalized CK values could be measured for the respective volunteer. With respect to specific organ toxicity, the elevated medical laboratory parameters were neither rated as an indication of direct liver and kidney damage caused by the exposition to UV 328 by the study investigators nor were the elevated CK values attributed to the exposure. Nevertheless, the extension of the study to additional subjects was suspended.

### Chemicals

UV 328 (purity 99.9%) was acquired from BASF SE. Reference substances of the metabolites 2-(2*H*-benzotriazol-2-yl)-4-(3-hydroxy-2-methylbutan-2-yl)-6-(*tert*-pentyl)phenol (UV 328-4/3-OH, purity 96%), 2-(2*H*-benzotriazol-2-yl)-6-(3-hydroxy-2-methylbutan-2-yl)-4-(*tert*-pentyl)phenol (UV 328-6/3-OH, purity 99%), 2-(2*H*-5-hydroxybenzotriazol-2-yl)-4,6-(di-*tert*-pentyl)phenol (UV 328-BT-OH, purity > 98%), 2-(2*H*-benzotriazol-2-yl)-4-(2-methylbutan-3-on-2-yl)-6-(*tert*-pentyl)phenol (UV 328-4/3-CO, purity 99%), 2-(2*H*-benzotriazol-2-yl)-6-(2-methylbutan-3-on-2-yl)-4-(*tert*-pentyl)phenol (UV 328-6/3-CO, purity 97.5%) and 2-(2*H*-benzotriazol-2-yl)-4-(2-methylbutan-3-on-2-yl)-6-(3-hydroxy-2-methylbutan-2-yl)phenol (UV 328-4/3-CO-6/3-OH, purity 95%) were custom synthesized in the Institute for Organic and Biomolecular Chemistry (Göttingen, Germany). Figure 1 shows the structures of the postulated metabolites.

Isotope labelled references of UV 328 and four metabolites were included as internal standards. 2-(4,5,6,7-^2^H_4_-2*H*-benzotriazol-2-yl)-4,6-di-*tert*-pentylphenol (UV 328-D4, purity 99,8%, isotopic purity 99.2%) was obtained from Angewandte Synthesechemie Adlershof (ASCA GmbH, Berlin, Germany). 2-(2*H*-benzotriazol-2-yl)-4-(1,1,1-^2^H_3_-3-hydroxy-2-(^2^H_3_-methyl)butan-2-yl)-6-(*tert*-pentyl)phenol (UV 328–4/3-OH-D6, purity > 95%, isotopic purity > 99%), 2-(2*H*-benzotriazol-2-yl)-6-(1,1,1-^2^H_3_-3-hydroxy-2-(^2^H_3_-methyl)butan-2-yl)-4-(*tert*-pentyl)phenol (UV 328–6/3-OH-D6, purity 95%, isotopic purity > 99%), 2-(2*H*-benzotriazol-2-yl)-4-(1,1,1-^2^H_3_-2-(^2^H_3_-methyl)butan-3-on-2-yl)-6-(3-hydroxy-2-methylbutan-2-yl)phenol (UV 328–4/3-CO-6/3-OH-D6, purity > 95%, isotopic purity > 99%) and 2-(2*H*-benzotriazol-2-yl)-4-(1,1,1-^2^H_3_-2-(^2^H_3_-methyl)butan-3-on-2-yl)-6-(*tert*-pentyl)phenol (UV 328–4/3-CO-D6, purity > 99%, isotopic purity > 98%) were custom synthesized by the Institute for Organic and Biomolecular Chemistry (Göttingen, Germany).

For enzymatic hydrolysis, β-glucuronidase/arylsulfatase from *Helix pomatia* was supplied from Roche Diagnostics GmbH (Mannheim, Germany). Ammonium acetate (for analysis), acetonitrile (ACN, for GC), ethanol (EtOH, absolute for analysis), dichloromethane (CH_2_Cl_2_, for analysis), formic acid (for analysis), sodium chloride (for analysis), trichloromethane (CHCl_3_, for GC) and toluene (for GC MS) were obtained from Merck KGaA (Darmstadt, Germany). Double distilled water was prepared using a milli-Q-system (Millipore, Bedford, USA). Sigma Aldrich (Steinheim, Germany) supplied *N*,*O*-bis(trimethylsilyl)acetamide with 5% TMCS (BSA + 5% TMCS) and 1-(trimethylsilyl)imidazole (TSIM) for derivatization. Human blood was donated by the principal investigator. Human CPD plasma was purchased by in.vent Diagnostica GmbH (Berlin, Germany). Both matrices were stored at refrigerator temperature.

### Analysis of UV 328 and its metabolites in blood

Analysis of blood samples was performed according to a previously published method (Denghel and Göen 2021). In summary, blood proteins and cellular components were precipitated by the addition of ACN. Afterwards, the supernatant was diluted with sodium chloride solution and the investigated analytes were extracted by dispersive liquid–liquid microextraction (DLLME) using a mixture of CH_2_Cl_2_ and ACN. The extracts were derivatized with BSA/TMCS and TSIM and finally measured via GC–MS/MS with advanced electron ionization (AEI). Limits of detection (LOD) were 0.1 µg/L and limits of quantification (LOQ) ranged from 0.2 to 0.4 µg/L for all analytes. Variation coefficients ranged from 2 to 9% for precision in series and from 3 to 11% for interday precision. Additionally, relative recovery rates between 80 and 100% were found.

#### Analysis of blood samples with hydrolysis

To determine the shares of analytes conjugated with glucuronic acid or sulfate, the analysis of blood samples was performed with and without hydrolysis for one volunteer. For hydrolysis, 1 mL of the blood sample was prepared in a 15 mL conical plastic tube (Sarstedt, Nümbrecht, Germany) with 200 μL of ammonium acetate buffer (1 mol/L, pH 6), 10 µL internal standard mix and 10 µL β-glucuronidase/arylsulfatase from *Helix pomatia*. The samples were then incubated at 37 °C over night. The further steps were performed according to the SOP.

#### Analysis of plasma samples

The method applied for the analysis of blood samples is described in detail in the supplemental material.

### Analysis of UV 328 and its metabolites in urine

The urine samples were processed according to a previously published procedure (Denghel and Göen 2020). In brief, urine samples were enzymatically hydrolyzed using β-glucuronidase/arylsulfatase from *Helix pomatia*. Afterwards, UV 328 and its metabolites were extracted by DLLME using a mixture of CHCl_3_ and EtOH. The extracts were derivatized with BSA/TMCS and TSIM and finally measured via GC–MS/MS with advanced electron ionization (AEI). Limits of detection (LOD) were 0.1 µg/L and limits of quantification (LOQ) ranged from 0.3 to 0.5 µg/L for all analytes. Variation coefficients ranged from 2 to 12% for precision in series and from 5 to 12% for interday precision. Furthermore, relative recovery rates between 90 and 110% were determined.

#### Analysis of urine samples without hydrolysis

The analysis of urine samples was performed with and without hydrolysis for one volunteer to determine the shares of analytes conjugated with glucuronic acid or sulfate. Initially, urine samples were thawed, equilibrated to room temperature and homogenized using a vortex mixer. Afterwards, the Monovettes^®^ were centrifuged for 2 min at 1600 *g* to sediment solid residue in the sample. 1 mL of the sample was transferred into a 15 mL conical tube (Sarstedt, Nümbrecht, Germany). Without hydrolysis, 1 mL of the urine samples was prepared with 200 μL of ammonium acetate buffer (1 mol/L, pH 6) and 10 μL of the internal standard mix solution. Afterwards, the urine samples were directly extracted according to the standard operating procedure (see Sect. “Analysis of UV 328 and its metabolites in urine”). Finally, the calculated analyte excretion rates (see Sect. “Evaluation of renal elimination”) obtained with hydrolysis were correlated with the ones prepared without hydrolysis for every detected compound. A linear curve fit was applied to the plotted data points. The resulting slope of the regression line described the percentage of free and therefore unconjugated analyte.

### Gas chromatography–tandem mass spectrometry analysis (GC–MS/MS)

The equipment and settings for the gas chromatograph and tandem mass spectrometer were previously described elsewhere for the analysis of UV 328 and its metabolites in both human blood and urine (Denghel and Göen 2020, 2021). A TRACE 1310 gas chromatograph, a TriPlus RSH autosampler and a TSQ 9000 triple quadrupole mass spectrometer with an AEI source installed (Thermo Fisher Scientific, Waltham, USA) were used for GC–MS/MS analysis. A volume of 1 μL sample was injected into the system in splitless mode. A 5%-phenyl-arylene/95%-dimethyl polysiloxane low-bleed capillary column (HP5 ms UI, 60 m × 250 μm × 0.25 μm) (Agilent Technologies, Santa Clara, USA) was used for gas chromatographic separation. Chromeleon Software Version 7.2.8 was used for device control and data analysis.

### Evaluation of blood kinetics

Deviating from the previously published method (Denghel and Göen 2021), the concentrations of UV 328 were determined through a quadratic calibration function. The kinetics of UV 328 and its metabolites in blood were ascertained by plotting average blood concentrations of all volunteers (in µg/L, arithmetic mean ± standard deviation) as function of time (in h). The ln-transformed mean excretion curve was plotted against the time (in h) to obtain the slope (*k*_el_, elimination rate constant) and the excretion half-life (*t*_1/2_) as follows:

$$t_{{1/2}} = ~\frac{{\ln \left( 2 \right)}}{{|k_{{{\text{el}}}} {\text{|}}}}.$$

Thereby, Microsoft Excel^®^ and Origin^®^ software were used for data processing and curve fitting, respectively.

#### Distribution of the analytes between blood compartments

Furthermore, the dispersion of UV 328 and its metabolites in the blood matrix was investigated using the related blood and plasma samples of one volunteer. Therefore, the concentrations of the analytes in whole blood and plasma were determined according to the SOP (Denghel and Göen 2021) and an established procedure (see supplemental material), respectively. The results for whole blood and plasma were plotted against the time points of the respective sampling and the plasma to blood ratios (*p/b*) were calculated by a simplified approach for estimating the AUC ratios. Therefore, all plasma concentrations (in µg/L) determined were added and divided through the sum of the respective blood concentrations (in µg/L) according to the equation:

$$p/b = {{\mathop \sum \limits_{{i~ = ~0}}^{n} c~\left( {{\text{plasma}}} \right)} \mathord{\left/ {\vphantom {{\mathop \sum \limits_{{i~ = ~0}}^{n} c~\left( {{\text{plasma}}} \right)} {\mathop \sum \limits_{{i~ =~0}}^{n} c~\left( {{\text{blood}}} \right)}}} \right. \kern-\nulldelimiterspace} {\mathop \sum \limits_{{i~ = ~0}}^{n} c~\left( {{\text{blood}}} \right)}}~.$$

The *p/b* ratio describes the concentration of the drug in whole blood compared to plasma and provides an indication of drug binding to erythrocytes (Kalamaridis and DiLoreto 2014). As whole blood consists of 55% plasma (Shier et al. 2018), approximately double concentrations of the analytes should be detected in the analysis of 1 mL plasma compared to 1 mL whole blood—provided that UV 328 and its metabolites do not bind to the erythrocyte fraction. Consequently, the expected *p/b* ratios of the investigated analytes should reveal values of approximately two to prove the availability of UV 328 and its metabolites in the plasma fraction.

### Evaluation of renal elimination

The analysis results of UV 328 and its metabolites in urine were obtained in µg/L. These concentrations were converted to the unit µg/h to calculate the excretion rate of each analyte for every time point using following equation:

$$R_{{E,i~}} = ~\frac{{c_{i} ~ \times ~V_{i} }}{{t_{i} - ~t_{{i - 1}} }}$$

Thereby, *R*_*E,i*_ is the renal excretion rate for the urine sample *i* (in µg/h), *c*_*i*_ is the analyte concentration in the urine sample (in µg/L), *V*_*i*_ is the volume of the urine sample (in L) and *t*_*i*_ and *t*_*i-*1_ are the elapsed time values (in h) of the sample and the previous sample, respectively.

Afterwards, the time course of the excretion rates *R*_*E,i*_ was recorded for the middle of the respective sampling period (*t*_*i,m*_ in h) according to the equation:

$$t_{i,m} = ~t_{{i - 1}} + ~\frac{{t_{i} - ~t_{{i - 1}} }}{2}.$$

The current excretion rates were plotted as a function of the average time of the respective sampling period for every volunteer and each analyte.

Finally, mean excretion curves were calculated by taking the average of related urinary sampling times and the corresponding renal excretion rates (in µg/h) for all volunteers’ samples (arithmetic mean ± standard deviation). The mean cumulative excreted amount (in µmol) of all volunteers was obtained by addition of each molar excreted amount:

$$\mathop \sum \limits_{{i~ = ~0}}^{n} \frac{{c_{i} ~ \times ~V_{i} }}{M}.$$

Thereby, *c*_*i*_ is the concentration of the metabolite in urine (in µg/L), *V*_*i*_ is the volume of the respective urine sample (in L) and *M* is the molar mass of the respective metabolite (in µg/µmol). Urinary excretion factors (*F*_UE_) were expressed as UV 328 dose equivalents in % to evaluate the total excretion of recovered UV 328 and its metabolites in urine after 24 and 72 h based on the applied oral dose of UV 328:

$$F_{{{\text{UE}}}} = ~\frac{{{\text{CE}}_{i} }}{{M_{D} }}~ \times ~100.$$

Thereby, CE_*i*_ is the mean cumulative amount of the respective metabolite (in µmol) and *M*_*D*_ is the mean dose of applied UV 328. Afterwards, excretion half-lives (*t*_1/2_) (in h) were ascertained as described in Sect. “Evaluation of blood kinetics” by plotting the current excretion rates as function of time.

Again, Microsoft Excel^®^ and Origin^®^ software were used for data processing and curve fitting.

## Results and discussion

### Identification of UV 328 metabolites and kinetics in blood

**Fig. 2:**
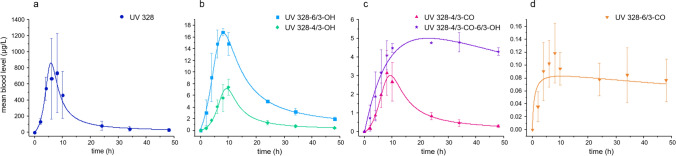
Kinetics of UV 328, UV 328-6/3-OH, UV 328-4/3-OH, UV 328-4/3-CO, UV 328-4/3-CO-6/3-OH and UV 328-6/3-CO in blood after oral exposure of three healthy volunteers to 0.3 mg UV 328/kg bodyweight (arithmetic mean ± standard deviation, *n* = 3)

The chromatograms of blood samples drawn before the oral exposure to UV 328 showed for all volunteers only small signals, which may relate to UV 328. However, they were of the same magnitude as those detected in the water blank. Consequently, these signals were assigned to contamination during sample preparation through applied devices or solvents. Nevertheless, their levels were extremely low (< LOQ) and negligibly for the study results. In contrast, none of the UV 328 metabolites were detected in the blood samples taken before the oral administration (< LOD). After exposure, UV 328 was found in the blood samples of all volunteers with a mean maximum value of 736 ± 489 µg/L at 8 h. Subsequently, a distinct decrease of UV 328 blood levels was observed to a mean level of 83.1 ± 54.2 µg/L after 24 h. Afterwards, the concentration of this parameter decreased slowly, leading to a final mean concentration of 29.1 ± 19.4 µg/L after 48 h (see Fig. 2a). The high standard deviations for the UV 328 levels 6–10 h after exposure are due to distinctly higher blood levels of the analyte in one of the volunteers compared to the other two. This might result from interindividual differences in adsorbing and distributing UV 328, possibly caused by different volumes of distribution in the human body—in spite of the body weight related application—as well as food effects and differences in the extent of first-pass metabolism in the intestine and liver (Derendorf et al. 2002; Derendorf and Schäfer 2011; Parkinson et al. 2010). Furthermore, five of the six investigated metabolites were detected in blood after oral exposure. For both hydroxylated metabolites, UV 328-6/3-OH and UV 328-4/3-OH, maximum levels of 16.9 ± 0.7 µg/L and 7.4 ± 0.9 µg/L after 8 and 10 h were calculated (see Fig. 2b). Among the oxo-metabolites, UV 328-4/3-CO was detected at maximum concentrations of 3.5 ± 1.4 µg/L 8 h post-exposure (see Fig. 2c). Thereby, these three metabolites showed distinct decreases in blood levels until 24 h after exposure to values of 5.2 ± 0.6 µg/L, 1.5 ± 0.5 µg/L and 0.9 ± 0.3 µg/L for UV 328-6/3-OH, UV 328-4/3-OH and UV 328-4/3-CO, respectively. The following decrease from 24 to 48 h was slower; however, were the analytes still quantifiable 48 h post-dosage.

In contrast, UV 328-6/3-CO was measured at LOD levels and was not reliably quantifiable. Nevertheless, a mean excretion curve could be plotted showing a plateau with concentrations around the LOD of 0.1 µg/L from 4 to 48 h after exposure (see Fig. 2d). A similar curve shape was revealed for the tertiary metabolite UV 328-4/3-CO-6/3-OH (see Fig. 2c). A steep increase was observed until 10 h post-dosage leading to a plateau between 4 and 5 µg/L until 48 h, indicating an extremely slow elimination of the analyte from blood. Oxidation at the benzotriazole moiety could not be demonstrated, as the metabolite UV 328-BTOH was not detected in any of the blood samples. This result was in line with the in vitro experiments previously published (Denghel et al. 2019). Thereby, both hydroxy and oxo-metabolites as well as the combined hydroxy and oxo-metabolite were verified in vitro, whereas oxidation at the benzotriazole (UV 328-BTOH) was not detected.

The results indicate that UV 328 may be resorbed in the intestine and further oxidized to metabolites with hydroxy and/or oxo functions. However, late maximum values of all detected analytes were revealed. Additionally, the transformation of UV 328 to its metabolites is rather slow and to a low extent. A possible explanation might be the special distribution of UV 328 in a multi-compartment model (Derendorf and Schäfer 2011). As UV 328 has a rather high molecular weight together with high lipophilicity (ECHA 2021a), it might be stored in lipid depots. Thus, it could be subjected to the enterohepatic cycle and, therefore, initially pulled out of the hepatic prompt metabolism temporary. Furthermore, the differently shaped elimination curve for UV 328-4/3-CO-6/3-OH might result from transformation of the tertiary metabolite out of the primary hydroxy metabolites UV 328-4/3-OH and UV 328-6/3-OH as well as the secondary metabolite UV 328-4/3-CO. Additionally, further resorption and intermediate storage in other compartments may be assumed.

Nevertheless, biliary excretion seems to be the major route of elimination for UV 328 and particularly the tertiary metabolite UV 328-4/3-CO-6/3-OH as biliary excreted substances might underlie the enterohepatic cycle which was assumed for other lipophilic xenobiotics, too (Höllerer et al. 2018). Therefore, UV 328 might be returned to the intestine with bile after passing the liver and yet again be resorbed, resulting in a late *t*_max_ of UV 328 and its metabolites in blood and extended elimination half-lives for all detected analytes (see Table 1). In addition, UV 328 and most of the quantifiable metabolites showed biphasic elimination curves where a faster elimination within 24 h was followed by a slower elimination afterwards. For the first elimination phase, elimination half-lives of 5.3, 8.3, 9.0 and 5.9 h were observed for UV 328, UV 328-4/3-CO, UV 328-6/3-OH and UV 328-4/3-OH, respectively, followed by 16.3, 16.4, 17.9 and 15.7 h from 24 to 48 h for the respective analytes. Resulting from the differently shaped elimination curve, the highest elimination half-life was calculated for UV 328-4/3-CO-6/3-OH with a value greater than 600 h.

**Table 1:** Characteristics of the kinetics of UV 328 and its metabolites in blood after oral exposure to 0.3 mg UV 328/kg body weight (mean value; *n* = 3) and the determined plasma to blood (*p*/*b*) ratios of the analytes (*n* = 1)

	*c*_max_ (µg/L)	*t*_max_ (h)	*t*_1/2_ (0–24 h) (h)	*t*_1/2_ (24–48 h) (h)	*p*/*b* ratio
UV 328	736.2 ± 488.7	8	5.3	16.3	1.7
UV 328-6/3-CO	< LOQ	–	–	–	–
UV 328-4/3-CO	3.5 ± 1.4	8	8.3	16.4	3.2
UV 328-6/3-OH	16.9 ± 0.7	10	9.0	17.9	2.3
UV 328-4/3-OH	7.4 ± 0.9	8	5.9	15.7	2.1
UV 328-BTOH	< LOD	–	–	–	–
UV 328-4/3-CO-6/3-OH	4.95 ± 0.72	24–34	< 600		2.0

To further investigate the metabolism and elimination of UV 328, possible shares of conjugated analytes in blood were determined. Therefore, blood samples of one volunteer were analyzed with and without addition of β-glucuronidase/arylsulfatase from *Helix pomatia*, incubation over night at 37 °C and further treatment according to the standard procedure previously published (Denghel and Göen 2021) (see Sect. “Analysis of blood samples with hydrolysis”). Afterwards, the calculated analyte concentrations with and without hydrolysis were plotted against the time points of blood samples. The resulting curves are shown in Figures S1 (supplemental material). For all detected parameters, similar curves were revealed for both treatments. Observed deviations were within the framework of measurement uncertainties. Consequently, UV 328 and its metabolites did not occur in blood as conjugates in significant share. Therefore, hydrolysis prior to the extraction and further treatment of the blood samples was not necessary to present their levels correctly.

### Distribution of UV 328 and its metabolites between blood compartments

In the study, a separate analysis of UV 328 and its metabolites in plasma was performed for one volunteer to estimate their distribution between the main blood compartments. The reliability data of the plasma analysis are displayed in the supplemental material. The resulting curves of the compared blood and plasma concentrations are shown in Figure S2. The curves demonstrate that the courses of the concentration in whole blood and in the plasma fraction were conform for all parameters. For the quantitative comparison of both matrixes the plasma to blood ratios were calculated based on a simplified AUC estimation procedure (see Sect. “Distribution of the analytes between blood compartments”). Table 1 displayed the calculated plasma to blood ratios. Thereby, values of around two were calculated for all analytes detected in both matrices, except for UV 328-4/3-CO for which a factor of 3.2 was revealed. However, this might be due to measurement uncertainties, as the *p*/*b* ratios could only be calculated for one volunteer. Furthermore, UV 328-4/3-CO was found in lower concentrations compared to the other quantifiable analytes. Nonetheless, all calculated plasma to blood ratios were in an acceptable range close to the reciprocal volume share of the plasma fraction. Consequently, it was proven that the plasma fraction was the exclusive blood compartment carrying UV 328 and its metabolites.

### Renal elimination kinetics of UV 328 and its metabolites

**Fig. 3:**
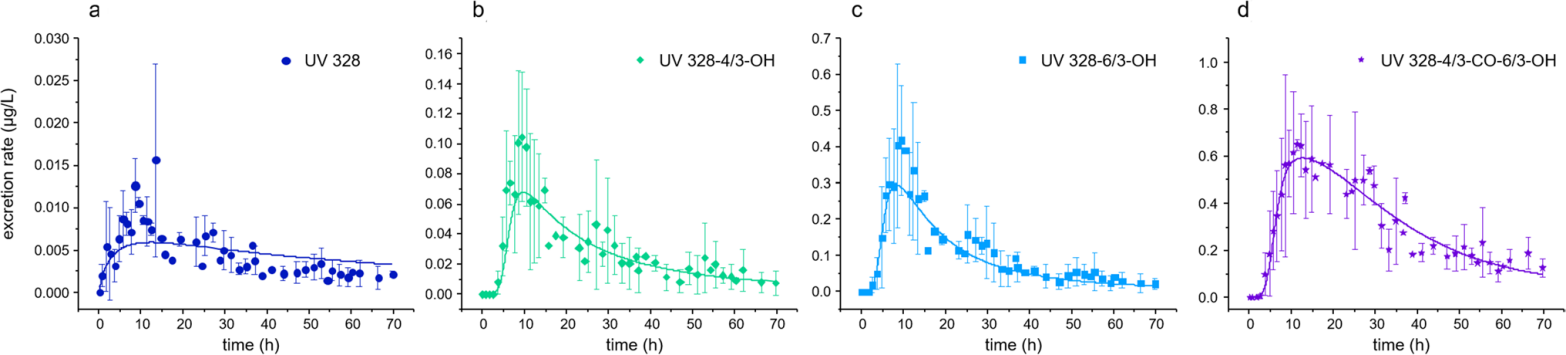
Kinetics of UV 328, UV 328-4/3-OH, UV 328-6/3-OH and UV 328-4/3-CO-6/3-OH in urine after oral exposure of three healthy volunteers to 0.3 mg UV 328/kg bodyweight (arithmetic mean ± standard deviation, *n* = 3)

None of the investigated analytes were detected in any of the urine samples drawn prior to UV 328 exposure (< LOD). Consequently, no background exposure as well as no contamination affected the study of the renal elimination kinetics. In contrast, UV 328 and four of its metabolites were detected in post-dosage urine samples. Thereby, UV 328 was found in very low excretion rates with a mean maximum value of 0.013 ± 0.003 µg/h for 8.5 h after exposure (Fig. 3a and Table 2). This is in line with the fact that renal excretion for UV 328 is rather unlikely due to its high molecular weight and elevated lipophilicity (Derendorf et al. 2002). Among the metabolites, low excretion rates were calculated for UV 328-4/3-CO. However, the mean excretion values for this parameter did not result in a comprehensible elimination curve. In general, this parameter was detected in very low concentrations for all volunteers. Depending on the volume and the creatinine content of the respective urine samples, the resulting excretion rates differed among the test persons and lead to high standard deviations for several mean excretion values of UV 328-4/3-CO (data not shown). In contrast, the primary hydroxy metabolites, UV 328-4/3-OH and UV 328-6/3-OH, were found in urine with mean maximum values of 0.10 ± 0.04 µg/h and 0.42 ± 0.15 µg/h after 9.3 h, respectively (Fig. 3b, c with Table 2). The tertiary metabolite UV 328-4/3-CO-6/3-OH was found with mean maximum excretion rates of 0.65 ± 0.02 µg/h 11.3 h after exposure (Fig. 3d and Table 2). UV 328-6/3-CO and UV 328-BTOH were not detected in the urine samples collected during the in vivo study, coinciding with the in vitro results previously published (Denghel et al. 2019).

**Table 2:** Characteristics of renal excretion kinetics of UV 328 and its metabolites after oral exposure to 0.3 mg UV 328/kg body weight (mean value; *n* = 3)

	RE_max_ (µg/h)	*t*_max_ (h)	*t*_½_ (h)	*F*_UE_ after 24 h (%)	*F*_UE_ after 72 h ≙ Fraction of oral dose (%)	Conjugation ratio (%)
UV 328	0.013 ± 0.003	8.5	56.7	0.001 ± 0.0001	0.001 ± 0.0001	16
UV 328-6/3-CO	–	–	–	–	–	–
UV 328-4/3-CO	–	–	–	0.003 ± 0.002	0.006 ± 0.005	100
UV 328-6/3-OH	0.42 ± 0.15	9.3	36.1	0.018 ± 0.003	0.027 ± 0.008	97
UV 328-4/3-OH	0.10 ± 0.04	9.3	46.4	0.004 ± 0.001	0.007 ± 0.003	100
UV 328-BTOH	–	–	–	–	–	–
UV 328-4/3-CO-6/3-OH	0.65 ± 0.02	11.3	53.3	0.038 ± 0.010	0.081 ± 0.018	100
Total				0.06 ± 0.1	0.12 ± 0.2	

*RE*_max_ maximum renal excretion rate (in µg/h) ± standard deviation, *t*_max_ time (in h) at which *RE*_max_ was found ± standard deviation, *t*_1/2_: elimination half-life of the respective metabolite (in h), *F*_UE_ mean urinary excretion factors as dose equivalents of UV 328 (in %) ± standard deviation after 24 and 72 h

Consequently, late maximum values for all quantifiable analytes were detected in urine after oral exposure. With higher oxidation of the analytes, the time span necessary to reach the maximum excretion rate extended. As a result, maximum peaks were calculated after 8.5, 9.3 and 11.3 h after exposure for the native compound UV 328, the primary metabolites UV 328-4/3-OH and UV 328-6/3-OH and the tertiary metabolite UV 328-4/3-CO-6/3-OH, respectively (Fig. 3 and Table 2). As the subsequent metabolite was formed based on its respective precursors, the limiting step seems to be the formation of the initial oxidation steps which may lead to a delayed maximum peak. This was accompanied by high elimination half-lives for the quantified analytes. Thereby, *t*_½_ values of 36.1, 46.4, 53.5 and 56.7 h were determined for UV 328-6/3-OH, UV 328-4/3-OH, UV 328-4/3-CO-6/3-OH and UV 328, respectively. While UV 328-4/3-CO-6/3-OH was still detectable in urine after the observed period of 72 h after exposure, none of the other quantified analytes was detected at this time point anymore. In contrast to blood where biphasic elimination was observed (see Sect. “Identification of UV 328 metabolites and kinetics in blood”), monophasic elimination kinetics were found in urine for the assessable analytes. This may be especially due to low analyte concentrations in the urine samples, and besides, interindividual variations between the volunteers preventing a clear distinction of different elimination phases.

In summary, the transformation of UV 328 to its metabolites occurred to be slow and to a low extent. Furthermore, low excretion rates prove the low renal elimination and indicate an alternative elimination pathway probably via feces. After oral ingestion, UV 328 might be subjected to the enterohepatic cycle and repeatedly released to the intestine with bile. The differently shaped elimination curve for UV 328-4/3-CO-6/3-OH in urine points out complex resorption and reabsorption processes. Thus, the determination of native UV 328 in blood might be the most promising option for human biomonitoring as renal excretion of UV 328 and its metabolites was revealed to be a minor elimination pathway.

Urinary excretion factors *F*_UE_ of UV 328 and its metabolites were calculated by the accounts of the parameters in all voids and were expressed as percentages of the applied oral dose. In total, 0.12% of the administered UV 328 dose was recovered as native substance and the identified metabolites in urine within 72 h after exposure (Table 2). The tertiary metabolite UV 328-4/3-CO-6/3-OH was identified as the urinary most prominent metabolite with a share of 0.081% of the oral dose, followed by UV 328-6/3-OH with 0.027%. UV 328-4/3-OH and UV 328-4/3-CO were found to be minor metabolites making up for 0.013% in summary of the applied dose within 72 h. Only 0.001% were excreted as native UV 328 via urine in 72 h. In the first 24 h, the total sum of excreted UV 328 and its metabolites made up for 0.06% of the applied oral dose. Between 24 and 72 h post-dosage further 0.06% of the administered UV 328 dose was excreted via urine.

In general, the total share of metabolites recovered in urine within 72 h after oral exposure is very low (~ 0.1%) leading to the question if further unidentified metabolites of UV 328 need to be investigated. As oxidations at the most reactive sites of metabolism were considered for the prediction of UV 328 metabolites (Denghel et al. 2019; Zhuang et al. 2017), the most likely metabolites were investigated. However, the intermediate dihydroxy metabolite was not taken into account for the current studies (Zhuang et al. 2017). Zhuang et al. performed in vitro experiments about the human metabolism of UV 328 using human liver microsomes and predicted this metabolite. Also, for a related substance, UV 327 (2-(5-chloro-benzotriazol-2-yl)-4,6-di-(tert-butyl)phenol), the formation of a dihydroxy metabolite was proven for in vitro experiments using human liver microsomes (Fischer et al. 2020). Thus, a dihydroxy metabolite is also very likely to be formed in the human body. However, as observed for the other primary and secondary metabolites, this parameter will probably be further oxidized to the tertiary UV 328-4/3-CO-6/3-OH metabolite.

Additionally, the regioisomer UV 328-6/3-CO-4/3-OH may be another potential metabolite of UV 328 which was not considered for these studies. However, as UV 328-6/3-OH is more dominant than UV 328-4/3-OH while UV 328-4/3-CO is preferably formed compared to UV 328-6/3-CO, the regioisomer UV 328-6/3-CO-4/3-OH might not be a UV 328 metabolite of great importance. As the turnover rate of the applied oral dose of UV 328 is extremely low for the investigated analytes in this study, it is more likely that UV 328 is not primary eliminated via urine but an alternative pathway. This supports the assumption of biliary excretion and elimination via feces as described for the kinetics in both blood and urine.

To further investigate the metabolism and elimination of UV 328, possible shares of conjugated analytes in urine were determined. Therefore, urine samples of one volunteer were analyzed with and without addition of β-glucuronidase/arylsulfatase from *Helix pomatia* and incubation according to the standard procedure previously published (Denghel and Göen 2020) (see Sect. “Analysis of urine samples without hydrolysis”). Without hydrolysis, only UV 328 and UV 328-6/3-OH were detected in very low concentrations, indicating that the residual metabolites detected are exclusively excreted as conjugates. Figure S3 shows the correlation of UV 328 and UV 328-6/3-OH parameters determined with and without hydrolysis. Thereby, a greater part of the calculated levels of UV 328 was below LOD, whereas only a few determined UV 328-6/3-OH concentrations where below LOQ and LOD, respectively. The slopes of the linear regression lines revealed values of 0.84 and 0.027 for UV 328 and UV 328-6/3-OH, respectively. Consequently, about 84% of UV 328 are excreted as native compound without conjugation to glucuronic acid or sulfate (Table 2). However, only about 3% of UV 328-6/3-OH are eliminated via urine without conjugation.

It can thus be concluded that the phenolic hydroxy moiety is difficult to access for conjugation due to sterically hindrance caused by both alkyl side chains of UV 328. In contrast, the investigated metabolites of UV 328 are excreted as glucuronide and/or sulfate conjugates. Further oxidation to hydroxyl functions facilitates the conjugation with glucuronic acid or sulfate, increases the water solubility and therefore enables the renal excretion. As a result, especially UV 328-4/3-OH, UV 328-6/3-OH and UV 328-4/3-CO-6/3-OH are the dominating metabolites detected in human urine samples. In contrast, the oxo-metabolites UV 328-4/3-CO and UV 328-6/3-CO were only detected at very low or even trace levels in urine, if at all. This emphasizes the importance of an additional hydroxy moiety for the conjugation with glucuronic acid or sulfate for an easy and effective renal elimination.

**Fig. 4:**
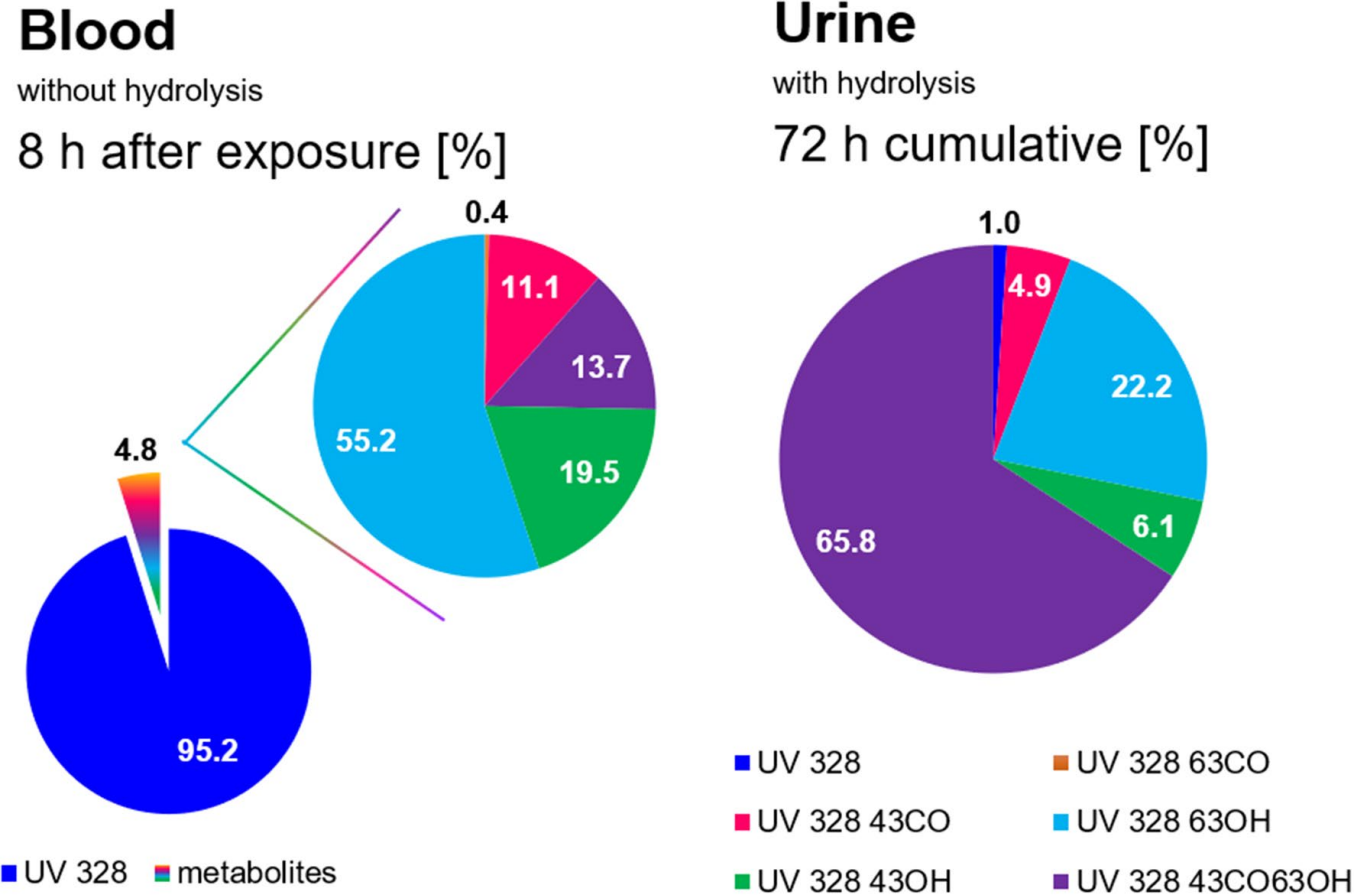
Comparison of the analyte shares in blood and urine (*n* = 3). Left: Shares of detected analytes in the blood samples taken 8 h after exposure and analyzed according to the SOP without hydrolysis. Right: Cumulative values within 72 h after exposure in urine samples analyzed according to the SOP with hydrolysis

When comparing the blood and urine results of the in vivo kinetics study about UV 328, only moderate differences become apparent (see Fig. 4). Most prominently, 95.2 ± 2.0% of the all detected parameters were found as UV 328 in the blood samples taken 8 h after the exposure. In contrast, only 1.0 ± 0.1% of the native compound were calculated for the cumulative amounts in urine within the total study duration of 72 h. Among the 4.8 ± 2.0% of quantified metabolites in blood, the highest shares were calculated for UV 328-6/3-OH (55.2 ± 6.0%), followed by UV 328-4/3-OH (19.5 ± 4.9%), UV 328-4/3-CO-6/3-OH (13.7 ± 2.5%) and UV 328-4/3-CO (11.1 ± 2.8%). In contrast, a shift towards the tertiary metabolite UV 328-4/3-CO-6/3-OH was observed for renal elimination. Among the cumulative amounts of all quantified analytes within 72 h, UV 328-4/3-CO-6/3-OH was detected with a total share of 65.8 ± 10.9%. Moreover, 22.2 ± 6.2% of UV 328-6/3-OH, 6.1 ± 2.3% of UV 328-4/3-OH and 4.9 ± 3.1% of UV 328-4/3-CO were calculated for the cumulative amounts of analytes detected within the total duration studied. With an increase in hydrophilicity caused by further oxidation, the elimination via urine after conjugation seems to be facilitated, which leads to UV 328-4/3-CO-6/3-OH as the predominant metabolite for renal excretion. In general, higher analyte concentrations were detected for blood samples compared to urine. Thereby, UV 328-BTOH was not detected in both matrices.

## Conclusion

With this study, the metabolism and excretion kinetics of UV 328, a benzotriazole UV absorber, were investigated in humans for the first time. The results proved the resorption and metabolism of UV 328 in the human body after oral exposure. Thereby, a significant resorption of UV 328 was revealed by high levels of the parent compound in blood. However, slow and low metabolism of UV 328 was demonstrated by targeted analysis in blood and urine. In total, only about 0.1% of the applied oral dose were recovered as UV 328 and its metabolites in urine within 72 h. The study results indicate that UV 328 may be stored in lipid depots and subjected to the enterohepatic cycle which may result in a late and repeated release to the intestine with bile and final preferred excretion pathway via feces instead of urine. Due to the minor relevance of the renal elimination and the slow kinetics, an accumulation of UV 328 and particularly of some of its metabolites may be expected for repetitive exposure scenarios.

## Supplementary Information

Below is the link to the electronic supplementary material.

Supplementary file1 (PDF 655 kb)
