# Supplementary material for: Human metabolism and kinetics of the UV absorber 2-(2H-benzotriazol-2-yl)-4,6-di-tert-pentylphenol (UV 328) after oral administration
Source: Arch Toxicol. 2021 Jun 27;95(8):2677–90. doi: 10.1007/s00204-021-03093-1 (PMC8298232; doi:10.1007/s00204-021-03093-1)
Supplement: Supplementary file 1 — Supplementary file1 (PDF 655 kb) [file 204_2021_3093_MOESM1_ESM.pdf]

Supplemental material

Analysis of plasma samples

For the analysis of plasma samples, the procedure for blood samples was adjusted as follows: Initially, CPD plasma was equilibrated to room temperature and homogenized by manual shaking. Frozen samples of the study participants were thawed, equilibrated to room temperature, and homogenized using a vortex mixer. Aliquots of 1 mL were prepared with 10 µL of the internal standard mix. Afterwards, the plasma proteins were precipitated by the additions of 3 mL ACN with 0.1 % formic acid. The samples were vortex-blended for 1 min and centrifuged for 15 min at 3000 x g. The supernatants were transferred into 25 mL tubes with conical bottom (Sarstedt, Nümbrecht, Germany) and diluted with 4 mL of 1 % sodium chloride solution. Afterwards, a mixture of 800 µL CH_2Cl_2 and 800 µL ACN was added to every tube. Again, the samples were vortex- blended for 1 min and centrifuged for 15 min at 3000 x g. The upper organic phases were transferred into 3 mL screw vials (Müller + Krempel AG, Bülach, Switzerland) and dried under nitrogen. The residues were derivatized with 90 µL BSA + 5 % TMCS. After 30 min reaction time at room temperature, 10 µL of 10 % TSIM were added. The samples were transferred to micro inserts, sealed with crimping caps, and incubated at 40 °C for 90 min. Finally, measurement was performed using GC-MS/MS with AEI ionization.

To prove the suitability and reliability of the procedure, important parameters such as limit of detection (LOD) and quantification (LOQ), relative recoveries and intraday precision were determined. Therefore, LOD and LOQ values of UV 328 and its metabolites were estimated using the signal-to-noise (S/N) ratio and calculated according to the following equations:

$$LOD\ [\mu g/L] = 3 * S/N\ ;\ LOQ\ [\mu g/L] = 10 * S/N$$

Thereby, LOD is three times the S/N ratio, while LOQ is ten times the S/N value. For the relative recoveries, two different concentrations (Q_{low} and Q_{high}) were investigated. For Q_{low} and Q_{high}, CPD plasma was spiked with 10 and 100 µg/L UV 328 as well as 2 and 10 µg/L of its metabolites, respectively. Both Q_{low} and Q_{high} were prepared in triple according to the established procedure. Afterwards, the mean relative recoveries (Rec_{rel}) were determined.

Therefore, the calculated concentrations were divided through the known, actual spiked concentrations and multiplied by 100:

$$Rec_{rel}\,[\%] = \frac{c_{calculated}\,[\mu g/L]}{c_{spiked}\,[\mu g/L]}\; x\; 100$$

For the intraday precision, both Q $_{low}$ and Q $_{high}$ were determined six times in one series. Afterwards, the resulting variation coefficients were calculated for quality assurance. The maximum tolerated value was 15 %.

Validation of the plasma analysis method

Validation of the plasma analysis method revealed LOD values of 0.1 µg/L or lower all analytes except UV 328-4/3-CO for which a limit of detection of 0.2 µg/L was revealed. The corresponding limits of quantification ranged between 0.1 and 0.6 µg/L. In summary, these values were comparable to the ones determined for the analysis of blood samples with LODs of 0.1 µg/L for all analytes and LOQs between 0.2 and 0.4 µg/L (Denghel and Göen 2021). Table S1 shows the calculated values for the relative recovery rates and the coefficients of variation for the precision in series for Q $_{low}$ and Q $_{high}$, respectively.

Table S1 Validation data of the plasma analysis method (estimated in human CPD plasma).

	Precision (n = 6)		Relative recovery (n = 3)	
Analyte	Q $_{low}$ [%]	Q $_{high}$ [%]	Rec $_{rel, low}$ [%]	Rec $_{rel, high}$ [%]
UV 328	14.7	3.8	99.2 ± 14.6	84.3 ± 3.2
UV 328-6/3-CO	6.9	4.3	92.9 ± 6.4	91.6 ± 3.9
UV 328-4/3-CO	6.1	14.2	96.6 ± 5.9	89.6 ± 12.7
UV 328-6/3-OH	2.3	4.5	100.1 ± 2.3	96.0 ± 4.3
UV 328-4/3-OH	7.6	6.7	104.9 ± 7.9	95.7 ± 6.4
UV 328-BTOH	5.5	13.1	104.3 ± 5.8	84.3 ± 11.1
UV 328-4/3-CO-6/3-OH	4.7	4.6	109.8 ± 5.1	99.7 ± 4.6

The coefficients of variation were below the tolerated maximum value of 15 % for all analytes and both investigated concentrations. Thereby, values lower than 8 % were calculated for all analytes and both concentrations except for Q $_{low}$ of UV 328 and Q $_{high}$ of UV 328-4/3-CO and

UV 328-BTOH, respectively. Consequently, excellent to good precision was proven for the plasma sample analysis procedure.

Furthermore, relative recovery rates between 80 and 110 % were detected. Thereby, UV 328 and UV 328-BTOH revealed the lowest recoveries with 84.3 and 89.6 % for Q_{high}. However, these values were still in the acceptable range between 80 and 120 % indicating suitable robustness of the method.

Supplemental material

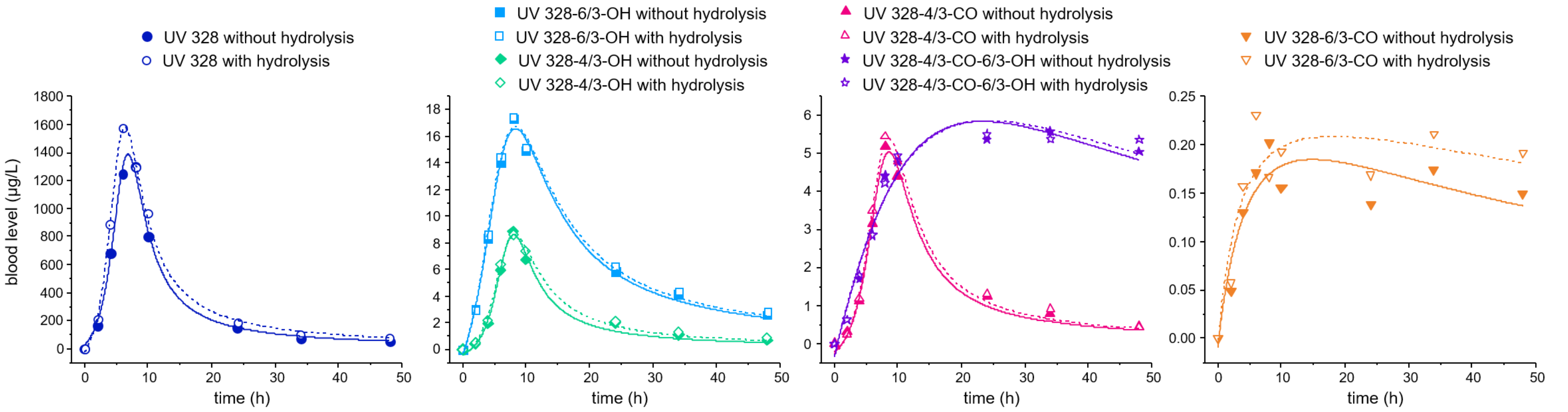

Figure S1 Kinetics of UV 328, UV 328-6/3-OH, UV 328-4/3-OH, UV 328-4/3-CO, UV 328-4/3-CO-6/3-OH and UV 328-6/3-CO in blood after oral exposure of one healthy volunteer to 0.3 mg UV 328/ kg bodyweight analyzed with and without hydrolysis.

Supplemental material

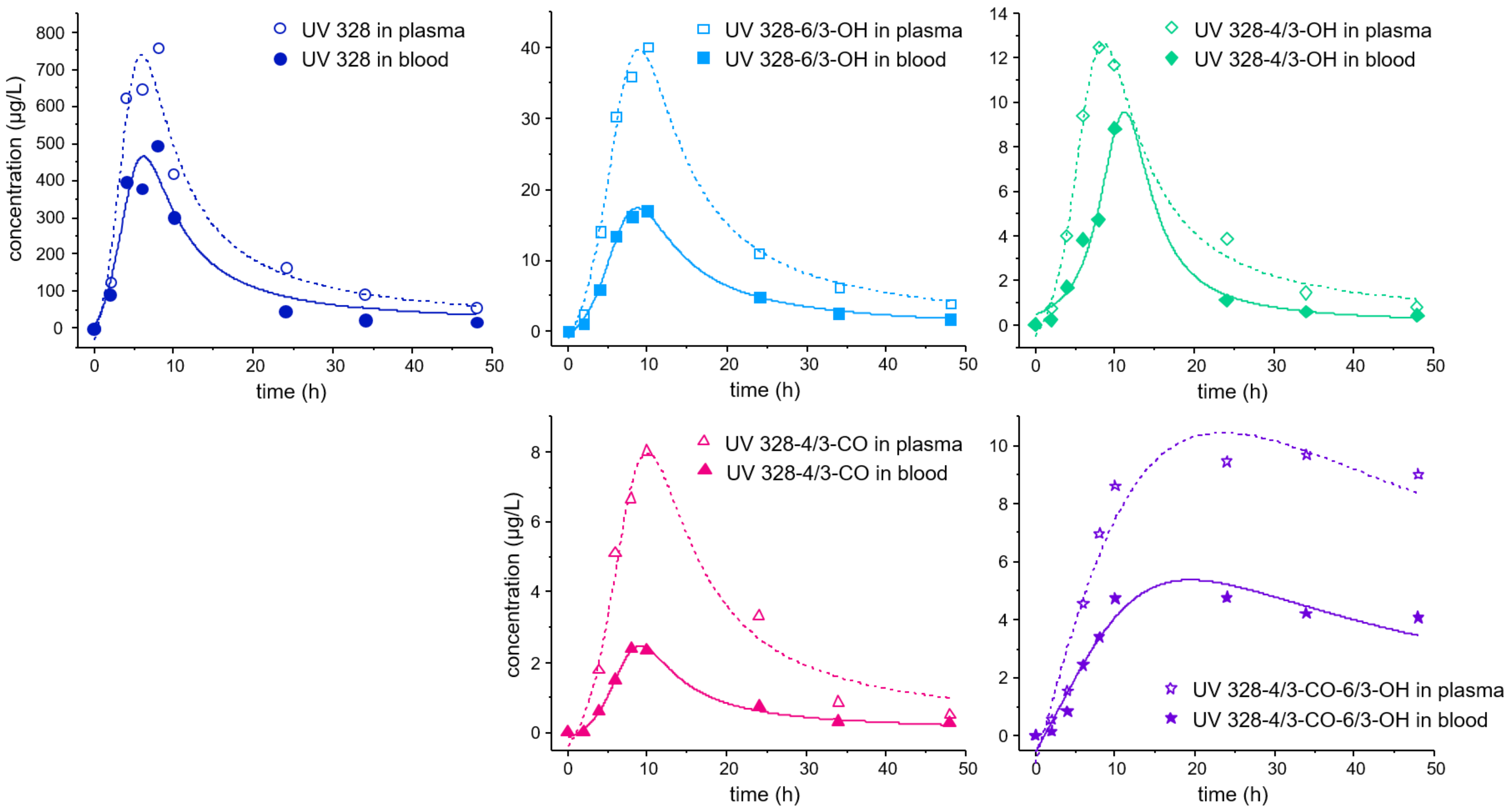

Figure S2 Kinetics of UV 328, UV 328-6/3-OH, UV 328-4/3-OH, UV 328-4/3-CO, UV 328-4/3-CO-6/3-OH and UV 328-6/3-CO in blood compared to plasma after oral exposure of one healthy volunteer to 0.3 mg UV 328/ kg bodyweight.

Supplemental material

a)

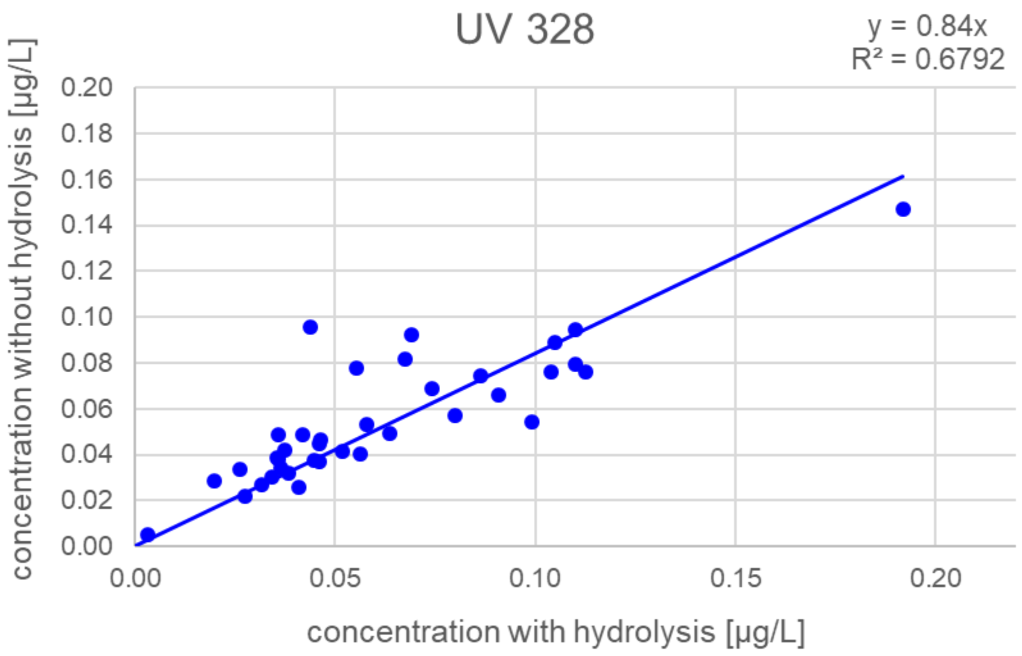

b)

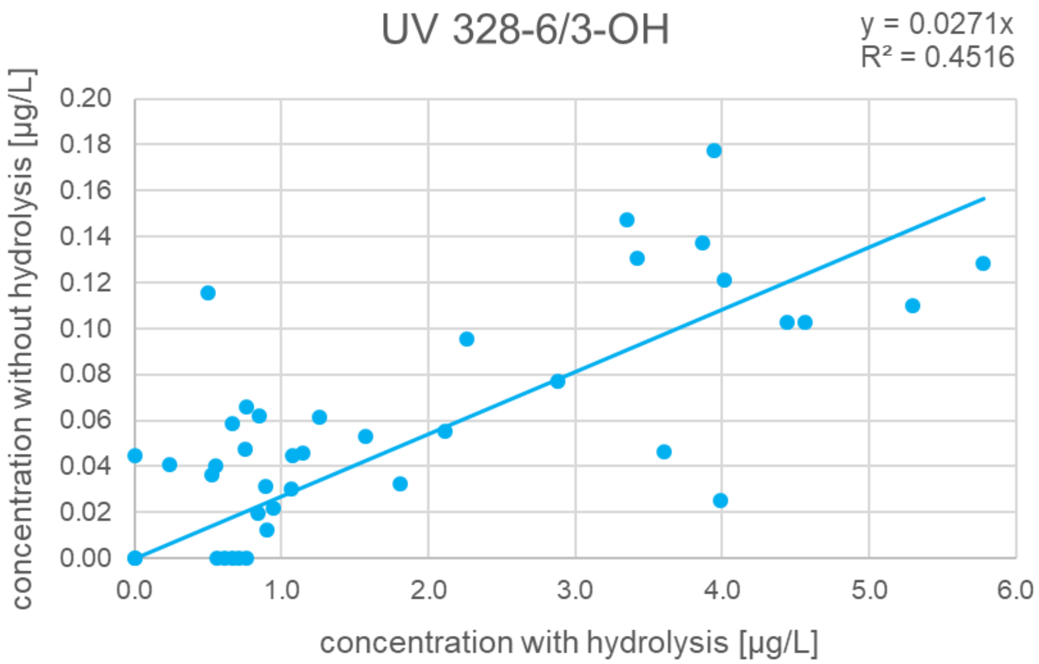

Figure S3 Correlations of the urine concentrations of UV 328 and UV 328-6/3-OH for the analysis with and without deconjugation of possible glucuronide of sulfate conjugates.

Supplemental material

References

Denghel H, Göen T (2021) Dispersive liquid-liquid microextraction (DLLME) and external real matrix calibration for the determination of the UV absorber 2-(2H-benzotriazol-2-yl)-4,6-di-tert-pentylphenol (UV 328) and its metabolites in human blood. Talanta 223:121699 doi:https://doi.org/10.1016/j.talanta.2020.121699
